# The Accuracy of Serum Biomarkers in the Diagnosis of Steatosis, Fibrosis, and Inflammation in Patients with Nonalcoholic Fatty Liver Disease in Comparison to a Liver Biopsy

**DOI:** 10.3390/medicina58020252

**Published:** 2022-02-08

**Authors:** Ivana Mikolasevic, Viktor Domislovic, Irena Krznaric-Zrnic, Zeljko Krznaric, Lucija Virovic-Jukic, Sanja Stojsavljevic, Ivica Grgurevic, Sandra Milic, Ivan Vukoja, Petra Puz, Merica Aralica, Goran Hauser

**Affiliations:** 1 Department of Gastroenterology, University Hospital Center Rijeka, 51000 Rijeka, Croatia; ikrznariczrnic@yahoo.co.uk (I.K.-Z.); smilic05@gmail.com (S.M.); goran.hauser@medri.uniri.hr (G.H.); 2 Faculty of Medicine, 51000 Rijeka, Croatia; 3 Department for Gastroenterology and Hepatology, University Hospital Center Zagreb, 10000 Zagreb, Croatia; viktor.domislovic@gmail.com (V.D.); zeljko.krznaric1@zg.ht.hr (Z.K.); 4 Faculty of Medicine, 10000 Zagreb, Croatia; lucija.jukic@gmail.com (L.V.-J.); ivica.grgurevic@zg.htnet.hr (I.G.); 5 Department of Gastroenterology, University Hospital Center “Sestre Milosrdnice”, 10000 Zagreb, Croatia; sanja.stojsavljevic1@gmail.com; 6 Department of Gastroenterology, Hepatology and Clinical Nutrition, University Hospital Dubrava, 10000 Zagreb, Croatia; 7 Department of Internal medicine, General Hospital Pozega, 34000 Požega, Croatia; iv.vukoja@gmail.com; 8 Department of Internal medicine, General Hospital Koprivnica, 48000 Koprivnica, Croatia; petra0307@gmail.com; 9 Clinical Institute for Laboratory Diagnostics, Clinical Hospital Centre, 51000 Rijeka, Croatia; merica.aralica@gmail.com

**Keywords:** nonalcoholic fatty liver disease, biomarkers, steatosis, fibrosis, inflammation

## Abstract

*Background and Objective*: This study was conducted to evaluate the diagnostic performance of various biomarkers for steatosis, fibrosis, and inflammation in comparison to a liver biopsy (LB) in patients with nonalcoholic fatty liver disease (NAFLD). *Materials and Methods:* This was a cross-sectional study that included 135 patients with biopsy-proven NAFLD. Fatty liver index (FLI), hepatic steatosis index (HSI), cell death markers (CK-18 M30 and CK-18 M65), FIB-4 index, NAFLD fibrosis score (NFS), BARD, and AST to platelet ratio index (APRI) were calculated and analysed. *Results:* FLI, HSI scores, and the cell death biomarkers showed poor diagnostic accuracy for steatosis detection and quantification, with an area under the curve (AUC) of <0.70. The cell death biomarkers likewise did not perform well for the detection of nonalcoholic steatohepatitis (NASH) (AUC < 0.7). As for the fibrosis staging, only APRI and the cell death biomarkers had moderate accuracy (AUC > 0.7) for advanced fibrosis, whereas FIB-4, BARD, and NFS scores demonstrated poor performance (AUC < 0.70). However, a combination of FIB-4 and NFS with the cell death biomarkers had moderate accuracy for advanced (≥F3) fibrosis detection, with an AUC of >0.70. *Conclusions:* In this first study on Croatian patients with NAFLD, serum biomarkers demonstrated poor diagnostic performance for the noninvasive diagnosis of liver steatosis and NASH. APRI and the cell death biomarkers had only moderate accuracy for diagnosing advanced fibrosis, as did the combination of FIB-4 and NFS with the cell death biomarkers. Further studies regarding serum biomarkers for all NAFLD stages are needed.

## 1. Introduction

Nonalcoholic fatty liver disease (NAFLD) is the most common chronic liver disease (CLD) that affects approximately one third of the population [1]. It has been shown that NAFLD is closely related to Type 2 diabetes mellitus (T2DM), obesity, and other components of metabolic syndrome (MetS) [1,2,3,4]. Nonalcoholic fatty liver (NAFL), or simple steatosis, represents the most common entity of the NAFLD spectrum that may progress to the necroinflammatory form, nonalcoholic steatohepatitis (NASH). Fibrosis in NAFLD can result in liver cirrhosis and hepatocellular carcinoma (HCC) development. In NAFLD patients, HCC can develop even in a non-cirrhotic liver [1,2,3]. Additionally, NAFLD may impose a huge economic burden since patients with NASH-related cirrhosis and HCC are becoming a growing group of candidates for liver transplantation [3]. NAFLD is not a “liver disease” only, but rather a multisystem disease that is associated not only with T2DM, chronic kidney disease (CKD), and cardiovascular diseases (CVD), but also with other extra-hepatic malignancies. According to the literature, the severity of NAFLD is directly related to those extrahepatic manifestations of NAFLD [5]. The presence of fibrosis is the most important “driver” associated with liver-related and overall mortality in NAFLD patients. According to study by Angulo P [6], an independent predictor of liver-related mortality was the fibrosis stage, starting from stage two (moderate) fibrosis [4,6]. Thus, the most important issue in NAFLD is the identification of patients at risk of advanced disease. What is important in terms of monitoring NAFLD progression is the differentiation of NASH from simple steatosis, detection of fibrosis, and differentiation of patients with no or minimal fibrosis to those with significant fibrosis, advanced fibrosis, or cirrhosis [1].

The diagnosis of NAFLD represents a clinical challenge in everyday practice since most patients are asymptomatic. The golden standard for the diagnosis and staging of NAFLD is still a liver biopsy (LB), which is an invasive method prone to sampling error. Regarding the significant economic burden of the disease and its potential consequences, there is a need for accurate, non-invasive, and cost-effective diagnostic methods [1]. In the last decade, numerous laboratory tests and biomarkers for steatosis, inflammation, and fibrosis detection, as well as imaging methods, are being intensively investigated.

Serum biomarkers such as NAFLD liver fat score, hepatic steatosis index (HSI), fatty liver index (FLI), etc. are used to detect or grade liver steatosis. The circulating keratin 18 (CK-18) fragments, M30 and M65, are cleaved during the period of cell death [7,8,9,10,11,12,13,14,15]. They represent good biomarkers of apoptosis and are among the most studied for distinguishing NASH from simple steatosis. Moreover, M65 detects both caspase-cleaved and uncleaved CK-18, and it is recognized as a marker of overall cell death (apoptosis and necrosis) [1,2,16]. In the published literature, M30 was more widely studied than M65 and showed moderate accuracy for the detection of NASH (66% sensitivity, 82% specificity) [7,8,9,10,11,12,13,14,15]. Circulating keratin 18 was investigated to detect not only NASH [7,16,17], but also fibrosis [7]. For the detection of fibrosis, some markers such as FIB-4 or BARD score were originally developed to detect the fibrosis stage in patients with viral hepatitis, while others were initially designed to predict fibrosis in NAFLD. One of these was the NAFLD fibrosis score (NFS). Models that we use today are much simpler. Some of them are based on the combination of clinical and biochemical variables, while others, such as the enhanced liver fibrosis (ELF) panel, are even more complex. Nowadays, the most investigated fibrosis biomarkers are the NAFLD fibrosis score (NFS) and FIB-4 [1,9].

The aim of our study was to evaluate the diagnostic performance of several serum biomarkers and their combinations for noninvasive diagnosis of liver steatosis, fibrosis, and inflammation (i.e., NASH) in patients with biopsy-proven NAFLD.

## 2. Materials and Methods

This cross-sectional study was conducted on a cohort of 164 patients with suspected NAFLD. Nonalcoholic fatty liver disease was suspected in patients with one or more MetS components (hypertension, T2DM, obesity, and/or dyslipidaemia), with a “bright” liver as seen on the abdominal ultrasound or with altered liver enzymes. The final diagnosis was established upon the results of LB that served as the reference method. Patients who signed an informed consent form and were older than 18 years were included in the study. Laboratory assessment was performed at the time of the biopsy.

Patients were recruited from the NAFLD clinic of the Gastroenterology Department of the University Hospital Centre Rijeka (UHCR) over a 26 month period (from October 2017 to December 2019). The primary outcome was to assess the diagnostic accuracy of various serum biomarkers and their combinations for diagnosing liver steatosis, fibrosis, and inflammation against LB serving as the reference method. The UHCR ethics committee approved this research (No.003–05/17–1/47). Appropriate informed consent forms were signed by all patients. We conducted the research in accordance with, and in agreement to, the International Conference on Harmonization guidelines on Good Clinical Practice and with the declaration of Helsinki.

### 2.1. Exclusion Criteria

**Figure 1:**
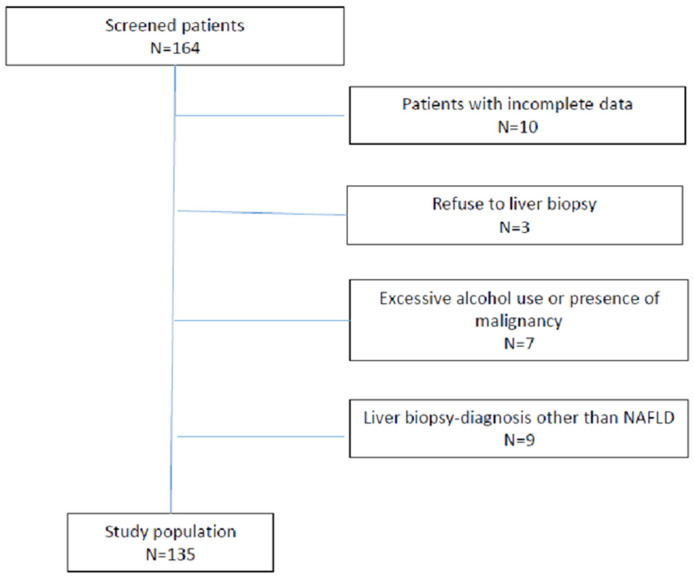
Flow chart of analysed patients.

In our study, we excluded patients with serology-confirmed chronic hepatitis B or C infection, those with a history of alcohol abuse (more than 20 g per day), and those who had used hepatotoxic medication within the last 6 months. In addition, exclusion criteria included a history of other metabolic, autoimmune, or cholestatic liver disease, known malignant disease, or clinical and laboratory indicators of damage to metabolic and synthetic liver function. Patients with a non-NAFLD disease as seen on liver biopsy findings (3 patients with primary biliary cholangitis, 3 patients with toxic hepatitis, 2 with autoimmune hepatitis, and 1 with intrahepatic cholestasis), as well as those with incomplete data, were also excluded, as seen in Figure 1.

### 2.2. Patient Characteristics

For each patient, we analysed demographic, clinical, and extensive laboratory characteristics that included exclusions of other CLD.

Serum biomarkers were calculated according to the following formulas:

- FIB-4 = (age (years) × aspartate aminotransferase (AST(IU/L))/(platelet count(10^9^/L) × ((alanin aminotransferase (ALT) (IU/L))1/2) [10]
- APRI = ((AST/ULN)/platelet count (10^9^/L)) × 100 [11]
- NAFLD fibrosis score: −1.675 + 0.037 × age (years) + 0.094 × BMI (kg/m^2^) + 1.13 × impaired fasting glycaemia or diabetes (yes = 1, no = 0) + 0.99 × AST/ALT ratio −0.013 × platelet (× 10^9^/litre) − 0.66 × albumin (g/dl) [12]
- BARD score was calculated as the weighted sum of the three variables (BMI > 28 = 1 point, AST/ALT ratio > 0.8 = 2 points, and diabetes = 1 point) [13]
- FLI = (e 0.953 ∗ loge (triglycerides) + 0.139 ∗ BMI + 0.718 ∗ loge (ggt) + 0.053 ∗ waist circumference − 15.745)/(1 + e 0.953 ∗ loge (triglycerides) + 0.139 ∗ BMI + 0.718 ∗ loge (ggt) + 0.053 ∗ circumference − 15.745) ∗ 100 [14]
- HSI = 8 × (ALT/AST ratio) + BMI (+ 2 if female; + 2 if diabetes mellitus) [15]

All scores were calculated from the blood samples taken at the time of the biopsy.

### 2.3. Liver Biopsy and Histological Analysis

Liver biopsies were performed under ultrasound guidance and according to a standardized protocol using the 16G Temno needle. Formalin fixation and paraffin embedding of the LB specimens was the first part of standardized histological protocol, followed by hematoxylin and eosin, as well as Mallory staining for fibrosis evaluation. The slices were analysed by pathologists experienced in the field and blinded to patient data. By using the NASH CRN scoring system and NAFLD activity score (NAS), steatosis (0–3), ballooning (0–2), lobular inflammation (0–3), and fibrosis (0–4) were evaluated [18]. To be qualified for the study, each patient had to have at least the presence of steatosis (>5% fatty transformed hepatocytes) in their liver histology findings. The final histological results were categorized as either NAFLD (non-NASH steatosis), NASH, or as other liver diseases if NASH criteria was not met or had overlapping issues (intrahepatic cholestasis, autoimmune hepatitis, toxic hepatitis, primary biliary cholangitis, or granulomatous liver disease). Severe pain that required observation and analgesia was the only post-liver-biopsy issue, as experienced by two patients.

### 2.4. M30 and M60 Analysis

Blood samples for both biomarkers were collected from the participants into plain tubes (BD Vacutainer^®^ tube, Plymouth, UK). Upon fast delivery, blood samples were centrifuged following serum separation from the cells. Collected samples were stored at −80 °C and thawed at room temperature before analysis in CDLD and UHCR.

### 2.5. M30-Enzyme-Linked Immunosorbent Assay (ELISA)

Serum concentrations of M30 were measured by the commercial ELISA kit, the M30 Apoptosense ELISA Kit (Peviva, VLVBIO, Nacka, Sweden), following the manufacturer’s instructions, and by the ELISA processor, the ThunderBolt (Gold Standard Diagnostics, Davis, CA, USA).

The ThunderBolt software calculated M30 results according to the calibration curve constructed from the M30 standard absorbances and their concentrations. The measuring range of the assay was 0–1000 U/L.

### 2.6. M65-Enzyme-Linked Immunosorbent Assay (ELISA)

Serum concentrations of M65 were measured by the commercial ELISA kit, the M65 EpiDeath ELISA Kit (Peviva, VLVBIO, Nacka, Sweden), following the manufacturer’s instructions, and by the ELISA processor, the ThunderBolt (Gold Standard Diagnostics, Davis, CA, USA).

The ThunderBolt software calculated M65 results according to the calibration curve constructed from the M65 standard absorbances and their concentrations. The measuring range of the assay was 0–5000 U/L. The cell death markers M30 and M65 were analysed at time of biopsy.

### 2.7. Statistical Analysis

Percentages were used for reporting categorical variables and medians with an interquartile range (25th and 75th percentiles) for reporting continuous variables. Receiver operating curve (ROC) and Yuden’s index were used to reveal the optimal values of investigated biomarkers for the diagnosis of fibrosis, steatosis, and NASH. Diagnostic performance of the combinations of biomarkers was also established using ROC analysis. All statistical analyses were performed using SPSS for Windows 22.0 (SPSS Inc., Chicago, IL, USA). A *p*-value of <0.05 was considered as statistically significant.

## 3. Results

The demographic, clinical, laboratory, and biopsy findings of the final cohort of 135 patients are depicted in Table 1.

### 3.1. Detection of Steatosis

The diagnostic accuracy of HSI and FLI scores for steatosis detection are shown in Table 2. Both of the investigated steatosis biomarkers showed poor accuracy for steatosis detection (Table 2). For M30, the diagnostic performance of discriminating different steatosis stages was as follows: S ≥ 2 AUC 0.65 (0.56–0.73) and S ≥ 3 AUC 0.64 (0.55–0.72). For M60, the corresponding values are S ≥ 2 AUC 0.66 (0.57–0.73) and S ≥ 3 AUC 0.70 (0.61–0.77). The values of the combination of the biomarkers M30 and M65 in detection of steatosis were S ≥ 2 AUC 0.65 (0.56–0.72) and S ≥ 3 AUC 0.70 (0.61–0.77). The values of the combination of HSI and M30 for the diagnosis of liver steatosis on biopsy were S ≥ 1 AUC 0.75 (0.66–0.82), S ≥ 2 AUC 0.74 (0.65–0.81), and S ≥ 3 AUC 0.67 (0.58–0.75). Furthermore, the combination of HSI and M65 performed as follows: S ≥ 1 AUC 0.74 (0.65–0.81), S ≥ 2 AUC 0.73 (0.64–0.80), and S ≥ 3 AUC 0.68 (0.59–0.76).

### 3.2. Detection of Nonalcoholic Steatohepatitis

We have analysed the diagnostic performance of M30, M65, and their combination in the detection of NASH. As demonstrated in Table 3, the cell death biomarkers could not adequately distinguish between simple steatosis and NASH.

### 3.3. Detection of Liver Fibrosis

The biomarkers M30, M65, and their combination had been assessed in evaluating their diagnostic performance for different fibrosis categories. The data on M30, M65, and the combination of M30 and M65 are depicted in Table 4, Table 5 and Table 6, respectively. Next, we have analysed four different indices (APRI, FIB-4, NFS, and BARD) and their diagnostic performance for different fibrosis grade categories. Complete data are shown in Table 7, Table 8, Table 9 and Table 10.

The combination of FIB-4 and M30 for the diagnosis of liver fibrosis on biopsy was F ≥ 1 AUC 0.56 (0.47–0.65), F ≥ 2 AUC 0.65 (0.58–0.75), and F ≥ 3 AUC 0.75 (0.66–0.82). Furthermore, the combination of FIB-4 and M65 performed as follows: F ≥ 1 AUC 0.55 (0.46–0.64), F ≥ 2 AUC 0.66 (0.57–0.74), and F ≥ 3 AUC 0.74 (0.66–0.81).

The combination of NFS and M30 for the diagnosis of liver fibrosis on biopsy was F ≥ 1 AUC 0.58 (0.47–0.68), F ≥ 2 AUC 0.69 (0.58–0.78), and F ≥ 3 AUC 0.73 (0.62–0.81). Furthermore, the combination of FIB-4 and M65 performed as follows: F ≥ 1 AUC 0.60 (0.49–0.70), F ≥ 2 AUC 0.68 (0.58–0.77), and F ≥ 3 AUC 0.75 (0.64–0.83).

## 4. Discussion

Two key questions regarding NAFLD patients are how to differentiate NASH from simple steatosis and how to detect advanced fibrosis, especially in the primary care setting [1,19]. This is the first study conducted on a Croatian population that attempted to independently validate several previously evaluated biomarkers of steatosis, fibrosis, and inflammation (i.e., NASH) over a cohort of patients with biopsy-proven NAFLD.

There is a need for adequate detection and quantification of steatosis since published data have shown the association of steatosis with extrahepatic complications such as T2DM, MetS, CVD, and CKD, as well as with some tumors [20,21,22,23,24,25]. Contrary to Fedchuk L [20], and according to the results of our population of NAFLD patients, FLI and HSI scores as well as the cell death biomarkers (M30 and M65) have shown poor diagnostic accuracy for steatosis detection and quantification. In the study by Fedchuk L [20], steatosis biomarkers were able to diagnose steatosis, but did not accurately quantify steatosis. These data suggest that imaging methods for steatosis detection (i.e., abdominal ultrasound) and quantification (i.e., controlled attenuation parameters) are still more accurate than serum biomarkers.

Early recognition of NASH is important for recognizing patients that are at higher risk of fibrosis development [1]. Since NASH is defined by specific pathohistological findings, LB is required for a conclusive diagnosis. In recent decades, many non-invasive methods have been investigated in terms of NASH detection. A study by Darweesh SK [7] included 135 participants with chronic hepatitis C infection (HCV), NAFLD, and a healthy liver, and found that serum CK-18 was significantly higher in the NAFLD group compared to the HCV and control groups. Additionally, CK-18 was significantly higher in NASH versus non-NASH patients [7]. Similarly, Joka D [16] had shown that both cell death biomarkers accurately differentiated patients with NASH from simple steatosis and healthy controls. However, their study population was very heterogeneous and included only 22 patients with NAFLD/NASH. Feldstein AE [26] analysed a cohort of 139 patients with biopsy-proven NAFLD and 150 age-matched healthy controls. According to this study, CK-18 fragments were significantly increased in NASH patients versus those without NASH, and they were an independent predictor of NASH [26].

In our study, we investigated the accuracy of CK-18 M30, CK-18 M65, and their combination in 135 patients with biopsy-confirmed NAFLD. Both biomarkers had poor accuracy for NASH detection (AUC < 0.7), although both (M30 and M65) had a high NPV for advanced fibrosis. Therefore, we suggest further investigation regarding the role and performance of these biomarkers in NASH.

Since significant fibrosis was recognized as an independent predictor of liver-related mortality [6], the identification of NAFLD patients with significant (F2) and advanced (F3) fibrosis is of great interest.

The major finding of our study was that only APRI and the cell death biomarkers M30 and M65, and their combination, had moderate accuracy (AUC > 0.7), with a good NPV (≥90%) for advanced fibrosis. Contrary to the previously published study by McPherson S [19], in our relatively large cohort of patients, FIB-4, BARD, and NFS scores did not show acceptable accuracy for fibrosis detection and grading (AUC < 0.70). However, the combination of FIB-4 and NFS with the cell death biomarkers M30 and M65 showed moderate accuracy for advanced (≥F3) fibrosis detection, with an AUC of more than 0.70 and a good NPV (>90%).

The data from our cohort suggest that the cell death biomarkers M30 and M65 may be useful in the primary care setting, alone or in combination with FIB-4 and NFS, to exclude advanced fibrosis in NAFLD patients. Similar results regarding these cell death biomarkers were obtained by other authors [16]. Since large numbers of NAFLD patients in Croatia are currently being referred to tertiary centres for specialist evaluation, the use of these non-invasive markers could substantially reduce the number of patients evaluated in the hospital setting [19]. NAFLD patients without fibrosis or with mild fibrosis (F1) are not expected to develop complications over a longer period of time (around 20 years), and thus could be adequately monitored at the primary care level. On the other hand, patients with advanced (≥F3) fibrosis should be monitored by a hepatologist. Further, NAFLD patients with stage F2 fibrosis are also at increased risk from liver-related and overall morbidity and mortality, especially in the presence of metabolic comorbidities, and should also be monitored by a hepatologist [4,6]. Our data suggest that the cell death biomarkers (M30 and M65) used for fibrosis detection are good markers for excluding advanced fibrosis, but their ability to exclude or detect significant fibrosis is in the “grey area” and still requires the application of additional diagnostic methods for accurate detection of at-risk patients.

Our study has some limitations. It was conducted in a tertiary care centre, which may represent a selection bias. Namely, in our cohort of patients, the MetS components were highly represented. Our patients were relatively obese, with a mean BMI of 32.3 kg/m^2^, 48% were diabetic, almost 74% of them had hypertension, and almost 73% had dyslipidaemia. Consequently, half of them had NASH, one third had advanced fibrosis, and almost half of them had significant fibrosis, which would be expected to be lower in the general population. Finally, in this study, we did not used a transient elastography (FibroScan) because the aim of this study was not a comparison of serum biomarkers with FibroSscan, but with LB. Further studies on this issue with larger number of patients are still required. However, the strengths of this study are its prospective enrolment and systematic biopsy confirmation.

## 5. Conclusions

In conclusion, in this first Croatian study with a relatively large cohort of patients with biopsy-proven NAFLD, we found that FLI and HSI scores, as well as the cell death biomarkers M30 and M65, show poor diagnostic accuracy for steatosis detection and quantification. The cell death biomarkers (CK-18 M35 and CK-18 M65) were not able to reliably differentiate NASH from simple steatosis. APRI and the cell death biomarkers (M30 and M65, as well as their combination) had moderate accuracy (AUC > 0.7), with a good NPV (≥90%) for diagnosing advanced fibrosis, while FIB-4, BARD, and NFS scores did not show acceptable accuracy for fibrosis detection and grading in our cohort. However, the combination of FIB-4 and NFS with the cell death biomarkers M30 and M65 showed moderate accuracy for the detection of advanced (≥F3) fibrosis. Our results suggest that the cell death biomarkers M30 and M65 may be used in the primary care setting, alone or in combination with FIB-4 and NFS indices, to exclude advanced fibrosis in NAFLD patients. Since NAFLD patients with stage 2 (moderate) fibrosis are also at increased risk of forming liver-related and overall morbidity and mortality, especially in the presence of metabolic comorbidities, they should also be identified and monitored by a hepatologist. Thus, further studies regarding these serum biomarkers for all NAFLD stages are required and eagerly awaited.

## Figures and Tables

**Table 1:** General characteristics of the analysed cohort of 135 NAFLD patients.

Variable (n = 135)	Value
Age, years (IQR)	59.3 (52–68)
Female gender, n (%)	65 (48.14)
BMI, kg/m^2^ (IQR)	32.3 (29.3–37)
Diabetes mellitus, n (%)	61 (45.1)
Arterial hypertension, n (%)	101 (74.8)
Hyperlipidaemia, n (%)	98 (72.6)
Platelet count, 10^9^/L (IQR)	222 (182–251)
AST, IU/L (IQR)	27.5 (22–38.5)
ALT, IU/L (IQR)	44 (28–57)
GGT, IU/L (IQR)	49 (26.5–82)
Alkaline phosphatase, IU/L (IQR)	72.5 (58–86)
Albumin, g/L (IQR)	44.4 (42–46.5)
Fasting glucose, mmol/L (IQR)	6.4 (5.6–7.8)
HOMA-IR score (IQR)	5.9 (3.8–8.6)
Waist circumference, cm (IQR) Male Female	111 (100–120) 112 (106–121) 104 (99–112)
Total cholesterol, mmol/L (IQR)	5.0 (4.2–6.0)
LDL cholesterol, mmol/L (IQR)	2.8 (2.1–3.7)
HDL cholesterol, mmol/L (IQR) Male Female	1.2 (1.1–1.4) 1.1 (1.0–1.4) 1.2 (1.1–1.4)
Triglycerides, mmol/L (IQR)	1.8 (1.3–2.5)
FLI (IQR)	92 (80.2–97)
HSI (IQR)	46 (41.8–49.8)
APRI (IQR)	0.48 (0.33–0.80)
FIB-4 (IQR)	1.24 (0.92–1.90)
NFS (IQR)	−0.88 (−2.1–0.03)
BARD (IQR)	2 (1–3)
M30 (IQR)	199 (74–258)
M65 (IQR)	259 (199–330)
Fibrosis stage, n (%) F0 F1 F2 F3 F4	29 (21.5) 49 (36.3) 32 (23.7) 17 (12.6) 8 (5.9)
Steatosis grade, n (%) S0 S1 S2 S3	5 (3.7) 57 (42.2) 40 (29.6) 33 (24.5)
NAS score, n (%) <5 ≥5	69 (51.1) 66 (49.9)

IQR, interquartile range; BMI, body mass index; AST, aspartate aminotransferase; ALT, alanin aminotransferase; GGT, gamma-glutamyl transferase; ALP, alkaline phosphatase; HOMA-IR, homeostasis model assessment-estimated insulin resistance; LDL, low-density lipoprotein; HDL, high-density lipoprotein; FLI, fatty liver index; HSI, hepatic steatosis index; APRI, AST to platelet ratio index; NFS, NAFLD fibrosis score; NAS, NAFLD activity score.

**Table 2:** The diagnostic accuracy of fatty liver index (FLI) and hepatic steatosis index (HSI) in the detection of steatosis.

	FLI		HSI	
	S ≥ S2	S = S3	S ≥ S2	S = 3
**cut-off**	**69**	**90**	**42.25**	**45.20**
**Se:**	25.53% (13.9%–40.3%)	64.0% (42.5%–82.0%)	87.01% (77.4%–93.6%)	78.12% (60.0%–90.7%)
**Sp:**	85.71% (71.5%–94.6%)	59.38% (46.4%–71.5%)	47.54% (34.6%–60.7%)	51.89% (42.0%–61.7%)
**PPV:**	66.7% (41.0%–86.7%)	38.1% (23.6%–54.4%)	67.7% (57.5%–76.7%)	32.9% (22.5%–44.6%)
**NPV:**	50.7% (38.6%–62.8%)	80.9% (66.7%–90.9%)	74.4% (57.9%–87.0%)	88.7% (78.1%–95.3%)
**LR+**	1.79 (0.7–4.3)	1.58 (1.0–2.4)	1.66 (1.3–2.1)	1.62 (1.2–2.1)
**LR−**	0.87 (0.7–1.1)	0.61 (0.3–1.1)	0.27 (0.1–0.5)	0.42 (0.2–0.8)
**AUC**	0.526	0.598	0.683	0.636
**SE**	0.062	0.068	0.046	0.054
**0.95 CI**	0.418–0.633	0.488–0.700	0.598–0.759	0.550–0.716

FLI, fatty liver index; HSI, hepatic steatosis index; Se, sensitivity; Sp, specificity; PPV, positive predictive value; NPV, negative predictive value; +LR, positive likelihood ratio; −LR, negative likelihood ratio; CI, confidence interval.

**Table 3:** The diagnostic performance of M30, M65, and their combination in the detection of NASH.

	M30	M65	M30 and M65
	NAS score ≥ 5	NAS score ≥ 5	NAS score ≥ 5
**Cut-off**	74	274	M30 = 74/M65 = 274
**Se:**	49.23% (36.6%–61.9%)	38.46% (26.7%–51.4%)	49.23% (36.6%–61.9%)
**Sp:**	85.71% (75.3%–92.9%)	94.20% (85.8%–98.4%)	85.51% (75.0%–92.8%)
**PPV:**	76.2% (60.5%–87.9%)	86.2% (68.3%–96.1%)	76.2% (60.5%–87.9%)
**NPV:**	64.5% (53.9%–74.2%)	61.9% (51.9%–71.2%)	64.1% (53.5%–73.9%)
**LR+**	3.45 (1.8–6.4)	6.63 (2.4–18.0)	3.40 (1.8–6.3)
**LR−**	0.59 (0.5–0.8)	0.65 (0.5–0.8)	0.59 (0.5–0.8)
**AUC**	0.683	0.662	0.691
**SE**	0.0378	0.037	0.039
**0.95 CI**	0.598–0.761	0.575–0.741	0.605–0.768

NAS, NAFLD activity score; Se, sensitivity; Sp, specificity; PPV, positive predictive value; NPV, negative predictive value; +LR, positive likelihood ratio; −LR, negative likelihood ratio; CI, confidence interval.

**Table 4:** The diagnostic performance of M30 for fibrosis grades greater than or equal to 1, greater than or equal to 2, and greater than or equal to 3.

	F ≥ F1	F ≥ F2	F ≥ F3
**cut-off**	**74**	**91**	**109**
**Se:**	32.40% (23.6%–42.2%)	46.43% (33.0%–60.3%)	66.67% (44.7%–84.4%)
**Sp:**	73.33% (54.1%–87.7%)	81.01% (70.6%–89.0%)	81.08% (72.5%–87.9%)
**PPV:**	85.1% (65.9%–91.4%)	63.4% (46.9%–77.9%)	43.2% (27.1%–60.5%)
**NPV:**	23.7% (15.5%–33.6%)	68.1% (57.7%–77.3%)	91% (84.5%–96.4%)
**LR+**	1.21 (0.6–2.3)	2.45 (1.4–4.2)	3.52 (2.2–5.7)
**LR−**	0.92 (0.7–1.2)	0.66 (0.5–0.9)	0.41 (0.2–0.7)
**AUC**	0.542	0.649	0.745
**SE**	0.045	0.042	0.057
**0.95 CI**	0.455–0.628	0.562–0.729	0.663–0.816

Se, sensitivity; Sp, specificity; AUC, area under the curve; PPV, positive predictive value; NPV, negative predictive value; LR+, positive likelihood ratio; LR−, negative likelihood ratio.

**Table 5:** The diagnostic performance of M65 for fibrosis grades greater than or equal to 1, greater than or equal to 2, and greater than or equal to 3.

	F ≥ F1	F ≥ F2	F ≥ F3
**cutoff**	**199**	**230**	**319**
**Se:**	29.52% (21%–39.2%)	37.5% (24.9%–51.5%)	58.33% (36.6%–77.9%)
**Sp:**	79.3% (60.3%–90.2%)	84.62% (74.7%–91.8%)	90.0% (82.8%–94.9%)
**PPV:**	83.8% (60%–93.8%)	63.6% (45.1%–79.6%)	56.0% (34.9%–75.6%)
**NPV:**	23.7% (15.7%–33.4%)	65.3% (55.2%–74.5%)	90.8% (83.8%–95.5%)
**LR+**	1.43 (0.7–3.1)	2.44 (1.3–4.5)	5.83 (3.0–11.2)
**LR−**	0.89 (0.7–1.1)	0.74 (0.6–0.9)	0.46 (0.3–0.7)
**AUC**	0.551	0.64	0.732
**SE**	0.043	0.04	0.057
**0.95 CI**	0.462–0.637	0.553–0.721	0.649–0.805

Se, sensitivity; Sp, specificity; AUC, area under the curve; PPV, positive predictive value; NPV, negative predictive value; LR+, positive likelihood ratio; LR−, negative likelihood ratio; CI, confidence interval.

**Table 6:** Diagnostic performance of the M30 and M65 combination for fibrosis grades greater than or equal to 1, greater than or equal to 2, and greater than or equal to 3.

	F ≥ F1	F ≥ F2	F ≥ F3
**cut-off**	M30 F1 = 74 M65 F1 = 199	M30 F2 = 91 M65 F2 = 230	M30 F3 = 109 M65 F3 = 319
**Se:**	13.33% (7.5%–21.4%)	46.43% (33.0%–60.3%)	66.67% (44.7%–84.4%)
**Sp:**	96.55% (82.2%–99.9%)	83.33% (73.2%–90.8%)	79.09% (70.3%–86.3%)
**PPV:**	93.3% (68.1%–99.8%)	66.7% (49.8%–80.9%)	41.0% (25.6%–57.9%)
**NPV:**	23.5% (16.2%–32.2%)	68.4% (58.1%–77.6%)	91.6% (84.1%–96.3%)
**LR+**	3.87 (0.5–28.2)	2.79 (1.6–4.9)	3.19 (2.0–5.1)
**LR−**	0.90 (0.8–1.0)	0.64 (0.5–0.8)	0.42 (0.2–0.7)
**AUC**	0.558	0.629	0.739
**SE**	0.051	0.046	0.06
**0.95 CI**	0.470–0.643	0.541–0.711	0.656–0.811

Se, sensitivity; Sp, specificity; AUC, area under the curve; PPV, positive predictive value; NPV, negative predictive value; LR+, positive likelihood ratio; LR−, negative likelihood ratio; CI, confidence interval.

**Table 7:** The diagnostic performance of APRI for fibrosis grades greater than or equal to 1, greater than or equal to 2, and greater than or equal to 3.

	F ≥ F1	F ≥ F2	F ≥ F3
**cut-off**	**0.4144**	**0.4639**	**0.5214**
**Se:**	60.61% (51.7%–69%)	70.89% (59.6%–80.6%)	79.49% (63.5%–90.7%)
**Sp:**	42.42% (25.5%–60.8%)	61.63% (50.5%–71.9%)	65.87% (56.9%–74.1%)
**PPV:**	80.8% (71.7%–88%)	62.9% (52.0%–72.9%)	41.9% (30.5%–53.9%)
**NPV:**	21.2% (12.1%–33.0%)	69.7% (58.1%–79.8%)	91.2% (83.4%-96.1%)
**LR+**	1.05 (0.8–1.5)	1.85 (1.4–2.5)	2.33 (1.7–3.1)
**LR−**	0.93 (0.6–1.5)	0.47 (0.4–0.7)	0.31 (0.2–0.6)
**AUC**	0.569	0.703	0.739
**SE**	0.051	0.042	0.047
**0.95 CI**	0.490–0.646	0.627–0.772	0.665–0.804

Se, sensitivity; Sp, specificity; AUC, area under the curve; PPV, positive predictive value; NPV, negative predictive value; LR+, positive likelihood ratio; LR−, negative likelihood ratio; CI, confidence interval.

**Table 8:** The diagnostic performance of FIB-4 for fibrosis grades greater than or equal to 1, greater than or equal to 2, and greater than or equal to 3.

	F ≥ F1	F ≥ F2	F ≥ F3
**cut-off**	**1.3895**	**1.5455**	**1.8137**
**Se:**	47.33% (38.5%–56.2%)	50.63% (39.1%–62.1%)	58.97% (42.1%–74.4%)
**Sp:**	81.82% (64.5%–93.0%)	81.18% (71.2%–88.8%)	84% (76.4%–89.9%)
**PPV:**	91.2% (81.8%–96.7%)	71.4% (57.8%–82.7%)	53.5% (37.7%–68.8%)
**NPV:**	28.1% (19.4%–38.2%)	63.9% (54.1%–72.9%)	86.8% (79.4%–92.2%)
**LR+**	2.60 (1.2–5.5)	2.69 (1.6–4.4)	3.69 (2.3–6.0)
**LR–**	0.64 (0.5–0.8)	0.61 (0.5–0.8)	0.49 (0.3–0.7)
**AUC**	0.634	0.638	0.68
**SE**	0.055	0.044	0.054
**0.95 CI**	0.556–0.708	0.559–0.711	0.602–0.705

Se, sensitivity; Sp, specificity; AUC, area under the curve; PPV, positive predictive value; NPV, negative predictive value; LR+, positive likelihood ratio; LR–, negative likelihood ratio; CI, confidence interval.

**Table 9:** The diagnostic performance of NFS for fibrosis grades greater than or equal to 1, greater than or equal to 2, and greater than or equal to 3.

	F ≥ F1	F ≥ F2	F ≥ F3
**cut-off**	**−1.8394**	**−1.6172**	**−0.0405**
**Se:**	78.31% (67.9%–86.6%)	83.72% (69.3%–93.2%)	52.38% (29.8%–74.3%)
**Sp:**	52.17% (30.6%–73.2%)	46.03% (33.4%–59.1%)	80.0% (69.9%–87.9%)
**PPV:**	85.5% (75.6%–92.5%)	51.4% (39.2%–63.6%)	39.3% (21.5%–59.4%)
**NPV:**	40.0% (22.7%–59.4%)	80.6% (64.0%–91.8%)	87.2% (77.7%–93.7%)
**LR+**	1.64 (1.1–2.5)	1.55 (1.2–2.0)	2.62 (1.5–4.7)
**LR−**	0.42 (0.2–0.7)	0.35 (0.2–0.7)	0.60 (0.4–0.9)
**AUC**	0.622	0.658	0.658
**SE**	0.069	0.055	0.075
**0.95 CI**	0.522–0.714	0.559–0.747	0.559–0.747

Se, sensitivity; Sp, specificity; AUC, area under the curve; PPV, positive predictive value; NPV, negative predictive value; LR+, positive likelihood ratio; LR−, negative likelihood ratio; CI, confidence interval.

**Table 10:** The diagnostic performance of BARD for fibrosis grades greater than or equal to 1, greater than or equal to 2, and greater than or equal to 3.

	F ≥ F1	F ≥ F2	F ≥ F3
**cut-off**	**1**	**2**	**3**
**Se:**	71.56% (62.1%–79.8%)	38.33% (26.1%–51.8%)	39.29% (21.5%–59.4%)
**Sp:**	51.72% (32.5%–70.6%)	62.82% (51.1%–73.5%)	82.73% (74.3%–89.3%)
**PPV:**	84.8% (75.8%–91.4%)	42.2% (30.5%–58.7%)	36.7% (19.9%–56.1%)
**NPV:**	32.6% (19.5%–48.0%)	57.0% (45.8%–67.6%)	84.3% (76.0%–90.6%)
**LR+**	1.48 (1.0–2.2)	1.03 (0.7–1.6)	2.27 (1.2–4.2)
**LR−**	0.55 (0.3–0.9)	0.98 (0.8–1.3)	0.73 (0.5–1.0)
**AUC**	0.666	0.591	0.636
**SE**	0.057	0.047	0.056
**0.95 CI**	0.580–0.744	0.504–0.674	0.549–0.716

Se, sensitivity; Sp, specificity; AUC, area under the curve; PPV, positive predictive value; NPV, negative predictive value; LR+, positive likelihood ratio; LR−, negative likelihood ratio; CI, confidence interval.

## Data Availability

Due to ethical reasons, the data are not available.
